# Psychology in the Nurse's Training

**Published:** 1920-02-14

**Authors:** 


### THE MATRONS' AND SISTERS' DEPARTMENT.

### PSYCHOLOGY IN THE NURSE'S TRAINING.

THE preparation of the mind for its future tasks is a recognised and most important part of the mental nurse's training. She will have to do with abnormal minds, and everyone grasps the fact that her own mind must be trained to meet and subdue harmful manifestations from the minds of her patients. The total absence of any mental preparation for ordinary medical and surgical work is accepted without question. Is that reasonable? Does it not leave nurses exposed to risk and helpless under many quite ordinary conditions in the sick-room?

It is hardly going too far to say that no patient's mind is entirely normal in acute illness. A febrile state upsets the equilibrium of the best-balanced individual. The shock of a severe accident affects the mind in a hundred different ways. The poison present in the blood of a rheumatic patient, the disease germs in the lungs of a consumptive, difficulties in breathing, difficulties in the action of the heart or kidneys, a sudden onslaught of pain or a long-continued ache all produce a strong mental effect. If we could read the thoughts of the sufferer we should probably find them clouded with apprehension. Not seldom we should detect strongly-marked delusions concerning his condition. A marked irritation of nerves or a tense resistance to the means employed for his cure are common features.

For lack of psychological training the private nurse is often a conspicuous failure. Instead of soothing she excites. She has never learned how to minister to the mind while tending the body, and her own undisciplined mind may often undo the good that her skilful fingers are accomplishing.

With the advent of the sister-tutor there is hope that the training of probationers may be envisaged as a whole, and that defects in their preparation for nursing the sick may be sought out and rectified. It is no arid study of the outlines of psychology which will enable the nurse to regard her patient as a suffering human being whose simpler mental needs she must study to meet. It is something at once more profound and less technical which is wanted. It is the admission into the training course of due recognition of the part played in the healing art by the patient's thoughts about disease and about the processes employed to make him well. The influence of the patient's subconscious impressions is now admitted as the principle upon which the modern hospital must be constructed. Why should not a bare whitewashed barn fitted with a skylight serve the doctor's purpose just as well as the sunny ward, gay with flowers and decorated to please the eye? Experience has proved that not a single detail is useless which helps the sick man to a sense of all being right with him, which soothes his mind and cheers his sub-conscious self. The next great step in hospital progress will be to set the nurses mentally in tune with the scheme of ward decoration. The difficult art of training the probationer's mind to carry about the sense of healing has already seen its beginnings. On its development depends the influence of the nurses of the future. The modern system of training, raised to its highest power, does induce this sixth sense in the elect. Can it be contended that the rank and file among probationers are aware of its existence?

There is another kind of mental training grievously lacking in nursing education. It is that which enables a woman to encounter disease, to live with it almost night and day, yet suffer no diminution of her own vitality, no haunting fear of succumbing to what she combats in her patients. It is a very real bogey, this, to imaginative women. Some live with a little skeleton of fear in their cupboard which peeps out whenever they are tired or worried. Tales of persons succumbing to "sympathetic" disease visit them in the night watches. They have had no advice or instruction in the art of repelling the enemy of morbid thoughts. They are too much ashamed of their secret fears to bring them out into the open and get them exorcised.

The simplest remedy, no doubt, for these morbid tendencies, which are far more common than is generally understood, is fresh air, exercise, outside interests. But however efficacious these may prove, there is need for a training of the mind lest in an unguarded hour the defences be broken down. The matron or sister-tutor who has learned to teach her nurses how to resist the morbid thoughts suggested by their daily companionship with disease and death will be able to reap her own reward in countless directions, not the least important in her own life and character.

